# Prevalence and *Toxoplasma gondii* Genotypes Circulating in Five Wild Corvid Species from Romania

**DOI:** 10.3390/pathogens14060572

**Published:** 2025-06-07

**Authors:** Călin Mircea Gherman, Anamaria Balea, Adriana Györke, Zsuzsa Kalmár, Angela Monica Ionică, Isabelle Villena, Furio Spano, Stéphane de Craeye, Vasile Cozma

**Affiliations:** 1 Department of Parasitology and Parasitic Diseases, University of Agricultural Sciences and Veterinary Medicine of Cluj-Napoca, Calea Mănăștur 3-5, 400372 Cluj-Napoca, Romania; calin.gherman@usamvcluj.ro (C.M.G.); anamaria.balea@usamvcluj.ro (A.B.); vasile.cozma@usamvcluj.ro (V.C.); 2 Parasitology Laboratory, Animal Health and Food Safety Agency Cluj Division, 400372 Cluj-Napoca, Romania; 3 Department of Microbiology, University of Agricultural Sciences and Veterinary Medicine of Cluj-Napoca, Calea Mănăștur 3-5, 400372 Cluj-Napoca, Romania; zsuzsa.kalmar@usamvcluj.ro; 4 Clinical Hospital of Infectious Diseases of Cluj-Napoca, 23 Iuliu Moldovan, 400348 Cluj-Napoca, Romania; 5 Laboratoire de Parasitologie, EA3800, IFR53, 51092 CHU Reims, France; ivillena@chu-reims.fr; 6 Centre National de Référence (CNR) Toxoplasmose/Toxoplasma Biological Resource Center (BRC), 51092 Reims, France; 7 Unit of Foodborne and Neglected Parasitic Diseases, Department of Infectious Diseases, Istituto Superiore di Sanità, 00161 Rome, Italy; furio.spano@iss.it; 8 Department of Quality of Laboratories, Belgian Scientific Institute of Public Health, Sciensano, Rue Juliette Wytsmanstraat 14, 1050 Brussels, Belgium; stephane.decraeye@sciensano.be

**Keywords:** *Toxoplasma gondii*, corvids, seroprevalence, molecular prevalence, genotype III

## Abstract

The apicomplexan parasite *Toxoplasma gondii* can potentially infect all warm-blooded animals, including birds, which, due to their high dispersal capabilities, are considered a significant candidate group of sentinel animals that reveal environmental contamination with this protozoan. In the present study, the serologic and molecular prevalences of *T. gondii* infection were determined in 333 corvids from Romania. Paired meat juice (n = 333) and heart samples (n = 244) were collected and analyzed using the modified agglutination test for antibodies, polymerase chain reaction (PCR) for DNA, and SAG2 molecular marker sequencing for genotyping. The overall *T. gondii* antibodies prevalence was 19.5%, with 48.1% infected jackdaws, 72.8% rooks, 89.7% hooded crows, 77.5% magpies, and 42.9% jays. Of 244 heart samples analyzed with PCR amplification, only 3 (1.2%) resulted positive and were shown to belong to genotype III through the sequencing of the SAG2 amplicon. This is the first extensive study on *T*. *gondii* in crows from Romania.

## 1. Introduction

Toxoplasmosis, caused by the apicomplexan *Toxoplasma gondii*, is one of the most widespread zoonoses globally [1]. This obligate intracellular parasite can infect all species of mammals, including humans and birds [2], while its ability to infect cold-blooded animals remains unproven [3]. Birds, feeding either on the ground, potentially contaminated with *T*. *gondii* oocysts, or as raptors–carnivores on prey carrying tissue cysts, are a candidate group of sentinel animals able to reveal environmental contamination with *T*. *gondii* oocysts [4].

The infection of birds with *T*. *gondii* is widely reported; numerous species from at least 15 orders, namely Accipitriformes, Falconiformes, Galliformes, Passeriformes, Columbiformes, Anseriformes, Charadriiformes, Struthioniformes, Strigiformes, Sphenisciformes, Ciconiiformes [5,6], Apodiformes [7], Suliformes, Phaethontiformes [8], and Gruiformes [9], are confirmed intermediate hosts of this parasite. Regarding domestic Galliformes, even though *T*. *gondii* does not pose a direct health risk to chickens, they represent an essential food safety issue due to the transmission risk associated with chicken meat consumption [10]. Additionally, *T*. *gondii*-infected free-range chickens that died from other causes and remained in the environment may constitute a contamination source for a range of bird species.

Among wild birds, the passerine family Corvidae members, which include ravens, crows, magpies, jays, treepies, choughs, and nutcrackers, are distributed worldwide except the southern tip of South America and the polar ice caps [11]. They are generalist species widespread in various environments, from harsh mountainous regions to plain ecosystems, but they have adjusted and are thriving in anthropogenically modified areas [12].

Corvids are considered omnivorous, opportunistic feeders [13,14]; their diet includes plants, animals, and miscellaneous foods. This wide variety of corvid food sources makes them susceptible to infection with various infective elements from the environment, such as eggs, oocysts, larvae, or via intermediate hosts of many parasites. Of these, *T*. *gondii* oocysts are reported in all matrices worldwide, specifically in water sources, soil from greenhouses, vegetable gardens, public parks, and other urbanized areas, industrial and commercial land, woodland, grassland, or around rubbish dumps [15]. Concomitantly, all these environments represent favorable areas for corvids nesting and feeding, thus exposing them to infection with *T*. *gondii* oocysts. Furthermore, *T*. *gondii* cysts are reported in the tissues of many living or dead organisms that enter the diet of corvids. Carrion meat [16], small mammals [17], insects [18], small reptiles, amphibians, mollusks [3], birds’ eggs [19], or slaughterhouse leftovers [20] are all potential sources of infection for corvids.

Given this background, this study aimed to serologically and molecularly evaluate the prevalence of *T*. *gondii* infection in birds of the Corvidae family.

## 2. Materials and Methods

**Figure 1:**
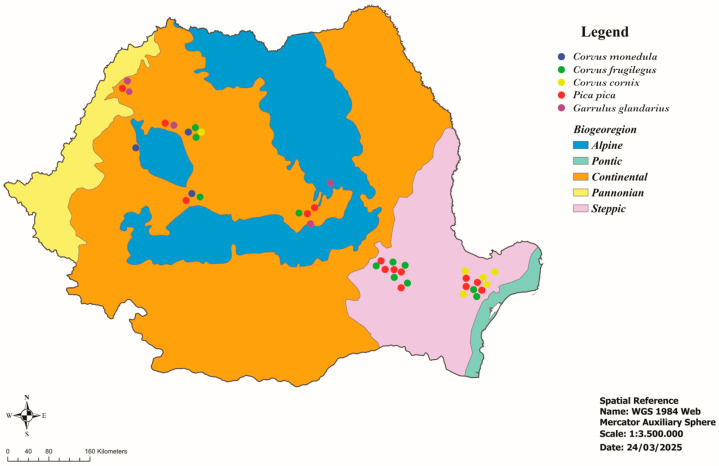
Geographical location of the examined corvids.

This study was performed on 333 corvids belonging to 5 species of the Corvidae family collected from all over Romania (Figure 1, Table 1). According to the Emergency Ordinance No. 7 of 6 March 2025, to amend and supplement Government Emergency Ordinance No. 57/2007 on the regime of protected natural areas, the conservation of natural habitats, and wild flora and fauna, the corvid species are not classified as protected species [21]. Therefore, most examined birds were hunted to combat the excessive number of resident corvids in certain areas or were found dead. The bird carcasses were collected, individually packaged, and frozen. The birds were identified based on morphological characters [22] and necropsied. During necropsy, the heart was collected and stored at −20 °C until processing. The meat juice samples obtained by freezing–thawing individual hearts were centrifuged at 5000 rpm for 5 min and stored at −20 °C until examined.

Meat juice samples were analyzed using the modified agglutination test (MAT) to detect anti-*T*. *gondii* antibodies using formalin-fixed tachyzoites (RH strain) (supplied by the National Reference Centre on toxoplasmosis/Toxoplasma Biological Resource Center from Reims, France) as antigens. The protocol was performed according to Villena et al. [23]. Samples with a titer ≥ 1:24 were considered positive [5,24].

Genomic DNA was extracted from heart samples (n = 244) using the commercial ISOLATE II Genomic DNA Kit (Bioline, London, UK). The extraction protocol was performed according to the manufacturer’s instructions. The extracted DNA was quantified with a NanoDrop 1000 Spectrophotometer (Thermo Fisher Scientific, Waltham, MA, USA) and stored at -20˚C until processing. DNA samples were analyzed using PCR for the detection of the 529 bp repetitive fragment using specific primers (Generi Biotech, Hradec Králové, Czech Republic), Tox4 (5′-CGCTGCAGGGAGGAAGACGAAAGTTG-3′) and Tox5 (5′-CGCTGCAGACACAGTGCATCTGG ATT-3′) [25]. For each reaction, amplification of DNA fragments was carried out in a final volume of 25 µL: 12.5 µL PCR Master Mix (2x Green PCR Master Mix, Rovalab, Teltow, Germany), 1 µL primer Tox4 (10 µM/µL), 1 µL primer Tox5 (10 µM/µL), 4 µL DNA and 6.5 µL ultrapure water. Positive (*T*. *gondii* RH strain) and negative (ultrapure water) controls were used in each set of reactions. The PCR inhibition was checked with serial dilution of selected PCR-negative samples (n = 10). Amplification was performed with a C1000TM Thermal Cycler (Bio-Rad, Berkeley, CA, USA). The amplification program was: 1 cycle of initial denaturation at 95 °C (1 min); 35 cycles of denaturation at 95 °C (15 s), annealing at 60 °C (15 s), and extension at 72 °C (10 s); 1 cycle of final extension at 72 °C (5 min). The PCR products were analyzed using electrophoresis in a 1.5% agarose gel stained with SYBR Safe DNA gel stain (Invitrogen, MA, USA) and visualized using the Gel Doc XR+ Gel Documentation System (Bio-Rad). The length of the amplified DNA fragments was estimated using a 100 bp molecular marker (O’GeneRuler 100 bp DNA Ladder, ready-to-use, Thermo Scientific). Positive DNA samples were amplified with primers specific for the SAG2 marker [26], purified using the commercial QIAquick PCR Purification Kit (Qiagen, Hilden, Germany), and sent for sequencing to Macrogen Europe (Amsterdam, The Netherlands). The obtained nucleotide sequences were compared with reference sequences from GenBank, using BLAST (Basic Local Alignments Tool) (https://blast.ncbi.nlm.nih.gov/Blast.cgi?PROGRAM=blastn&PAGE_TYPE=BlastSearch&LINK_LOC=blasthome, accessed on 15 April 2025) analysis [27].

Statistical data processing was performed with Microsoft Excel and Epi Info programs [28]. Frequency, prevalence, and 95% confidence interval were calculated. Differences between prevalences were statistically analyzed using the Chi-square test. The *p*-value < 0.05 was considered statistically significant.

## 3. Results

The overall prevalence of anti-*T*. *gondii* antibodies was 19.5% (65/333; 95% CI: 15.6–24.1) with MAT (Table 2). Depending on the species, the prevalence was 9.6% in jackdaw (5/52), 22.6% in rook (44/195), 28.2% in hooded crow (11/39), 12.5% in magpie (5/40), whereas no infected jays were found. The maximum dilution at which anti-*T*. *gondii* antibodies were detected was 1:192 in rooks (Table 3). The differences in seroprevalence recorded between corvid species were statistically significant (*p* = 0.0001).

The molecular prevalence was lower, with *T*. *gondii* DNA detected in three (1.2%; 95% CI: 0.25–3.55) of the 244 heart samples analyzed. The positive samples originated from the rook (1/106), the hooded crow (1/39; MAT ≥ 1:24), and the magpie (1/40). Two of three PCR-positive samples originated from birds with antibody titers below the cut-off value (rook MAT ≥ 1:12, magpie MAT ≥ 1:6). The differences between the prevalences recorded by species were not statistically significant (*p* = 0.75).

Sequence analysis of the three SAG2 amplicons showed 100% homology to genotype III (VEG strain). The sequence was deposited in GenBank under the accession no. PV485377.

## 4. Discussion

Corvids are one of the most abundant and extensively studied and abundant groups of passerine birds worldwide [29]. The populations of the species incorporated in the present study include millions of adult specimens worldwide, with a stable trend (Table 4) [30,31]. The abundance of corvid populations, their varied diet and sedentarism, the synanthropization favorable to their development, and implicitly, their close contact with humans or domestic animals make these birds a possible reservoir of various pathogens, some with zoonotic potential, including the apicomplexan *T*. *gondii*. Multiple studies on the prevalence of infection in corvids have been conducted worldwide.

In Asian countries, *T*. *gondii* infection is reported in several corvid species. In Iran, a molecular prevalence of 16.36% (9/55) was recorded by studying the brain tissue of hooded crows, revealing the same genotype III in eight isolates [32]. Through histopathological examination of brain tissue, *T*. *gondii* was identified in 3.8% of 125 rooks from the same country [33]. An increased molecular prevalence of 34% (34/100) was recorded from the brains of Eurasian magpies collected from Northwestern Iran; 5.9% of the isolates belonged to *T*. *gondii* genotype II and 94.1% to type III [34]. In Kazakhstan, the infection was histopathologically confirmed, recording a prevalence of 1.7% in carrion crows (*Corvus corone*) [35]. In Western Asia, the infection was reported in carrion crows from Turkey, with molecular prevalence determined from the brain tissue of 4.7%; still, the infection was not confirmed in the single magpie specimen analyzed [36]. In the same area, *T*. *gondii* seroprevalence was studied in crows in Israel to assess exposure to this pathogen in scavenger birds and their possible role in the epidemiology of toxoplasmosis [37]. Using MAT, 52 of the 122 examined crows (42.6%) were seropositive. By species, the infection was seroconfirmed in 48 of 101 carrion crows (47.5%), 2/5 (40%) jackdaws, and 2/16 (12.5%) house crows (*Corvus splendens*), a common bird of Asian origin currently spread in many parts of the world. PCR analysis of brain-extracted DNA from a crow was positive and identified as genotype II. The seroprevalence of *T*. *gondii* infection, determined by the latex agglutination test, was 35% in house crows in Pakistan; the higher seropositivity might be due to the scavenging of infected carrion [38].

The first report of *T*. *gondii* infection in corvids worldwide originated in New York State, USA [39]. Parasites were identified from the brain tissue of the American crow (*Corvus brachyrhynchos*) by intraperitoneal passages in mice. The strain isolated from this crow species was less pathogenic to mice than others and highly pathogenic to rabbits, while ducklings seemed refractory. However, the incidence of natural infection seems low (around 1%). More recently, however, infection in the same corvid species was not confirmed in Colorado, probably because only two specimens were examined [40]. *T*. *gondii* infection was also immunohistochemically diagnosed in the American native ‘Alala (*Corvus hawaiiensis*) species, the most endangered corvid in the world, in Hawaii [41]. Also, in America, the infection was studied in Mexico in the common raven (*Corvus corax*), one of the most widespread corvid species in the Northern Hemisphere; still, the infection was not serologically confirmed, probably due to the limited number of birds examined [42].

The majority of the studies on *T*. *gondii* infection in corvids were carried out on the European continent. In Western Europe, the infection was molecularly confirmed in Spain in magpies (5/33; 15.1%) and jays (5/23; 21.7%) from the brain and serologically with MAT in common ravens (91/113; 80.5%) but not in Eurasian jackdaws [4,24,43]. In Portugal, it was proven in carrion crows (1/3; 33.3%) [7], whereas in Italy, magpies (41/651; 6.3%) and hooded crows (4/120; 3.3%) were serologically and molecularly confirmed hosts, infected with either genotype II or III; in contrast, the rook and Eurasian jay did not reveal *T*. *gondii* infection [44,45]. In Central and Eastern Europe, studies of *T*. *gondii* parasitism in birds are generally scarce, and regarding crows, the infection was reported in only two countries. In the Czech Republic, jackdaws (1/5; 20.0%), rooks (89/495; 18.0%), and jays (1/43; 2.3%) were confirmed as *T*. *gondii* hosts [9]. In Serbia, molecular analysis of the hearts showed that 40% of rooks and 35.3% of hooded crows were infected with *T*. *gondii*, indicating substantial exposure to the parasite, and suggesting a high level of environmental oocyst contamination [46].

In Africa, although several studies on *T*. *gondii* infection in birds exist, they do not include corvids. However, it has been molecularly confirmed in bucerotids (*Tockus leucomelas*), columbids (*Spilopelia senegalensis* and *Streptopelia semitorquata*), and larids (*Larus michahellis*) [47,48].

Here, we report on *T*. *gondii* infection in corvids from Romania, revealing variable prevalences in all examined species. The differences with some of the previous studies may be due to the diagnostic method employed, i.e., serology, molecular testing, or histopathology. Among the serological methods, the most commonly used in the diagnosis of toxoplasmosis are the indirect hemagglutination test (IHA), the immunofluorescence antibody test (IFAT), and the modified agglutination test (MAT). The literature has shown the MAT to be specific and sensitive for testing bird antibodies against *T*. *gondii,* with the additional advantages of not requiring special equipment or species-specific conjugates [5,49]. In molecular testing, false results may arise due to the type of tissue used for DNA extraction. The brain seems to be the predilection organ of *T*. *gondii* in mammals, as confirmed in pigs, sheep, and rodents [50,51,52]. It is followed by the heart, lungs, skeletal muscles, diaphragm, liver, and kidneys [53,54,55,56,57]. However, in birds, the heart, brain, or liver are equally considered predilection organs, as demonstrated in experimental or natural infections in gallinaceous and anseriforms [58,59,60,61]. Additionally, the inconsistency between the prevalence revealed using MAT and PCR is explainable by the different targets of these methods. The MAT detects specific IgG antibodies, which may persist for months or even years, suggesting prior exposure to the infection. In contrast, PCR detects the *T*. *gondii* DNA in tissues, where the parasite load can be low and not uniformly distributed.

**Figure 2:**
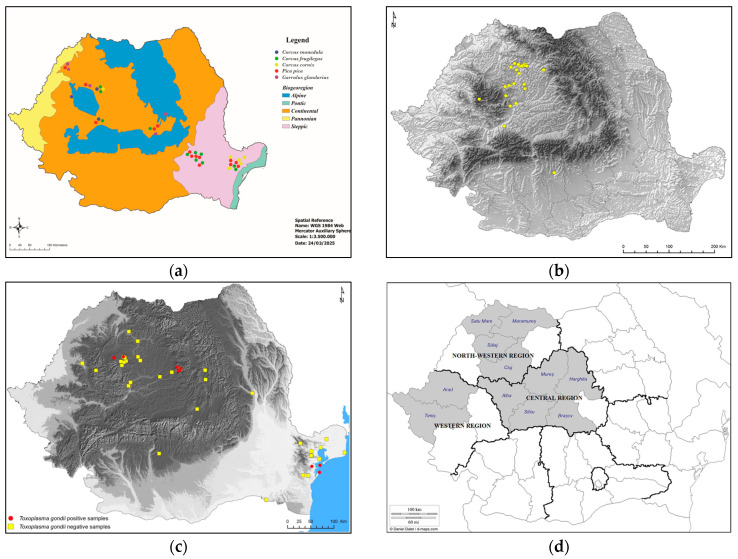

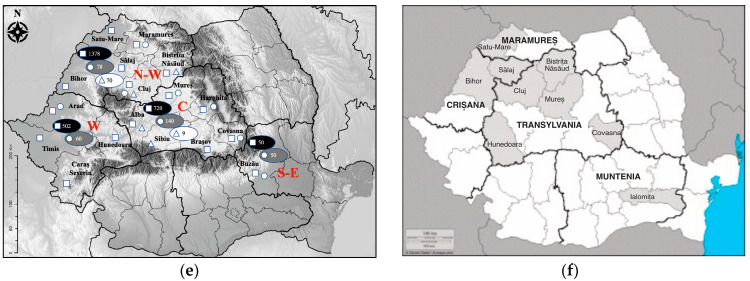
Central and northwestern locations of most studies targeting *T*. *gondii* infection prevalence in definitive and intermediate hosts in Romania: (**a**) Crows—current study; (**b**) cats [66]; (**c**) small mammals [17]; (**d**) pigs [68]; (**e**) sheep (squares—counties of sheep origin; circles—counties of lambs origin; triangles—counties of sheep abortions origin; black ovals—total number of sheep per region; gray ovals—total number of lambs per region; white ovals—total number of sheep abortions per region) [69]; (**f**) goats [70].

**Figure 3:**
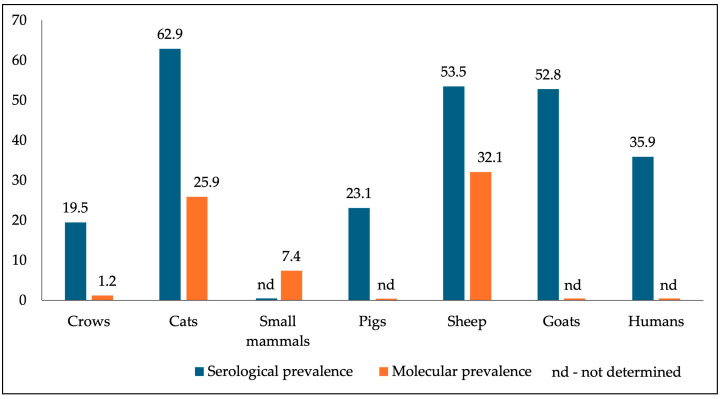
The current study’s *T. gondii* infection sero- and molecular prevalence in crows corroborated with that reported in domestic definitive and intermediate hosts.

Regardless of the methods and tissues used, the pooled prevalence of *T*. *gondii* infection in crows has been systematically reviewed and reached 25% with limits between 7 and 49%; this is amongst the highest values recorded in wild birds, exceeded only by owls (29%; 18–42%), gulls (29%; 12–49%), kestrels (25%; 20–30%), and hawks (25%; 15–36%) [6]. This increased prevalence of *T*. *gondii* in corvids is influenced by several risk factors. The data reported indicate that epidemiological variables, such as environmental conditions, diet, feeding behavior, and, implicitly, the trophic level to which they belong, age, sedentary habit, the crows’ population density, the season, habitat, and the density of animal, human, and feline populations in the area can influence the prevalence of *T*. *gondii* in wild birds [4,37,44,62,63,64]. Out of the mentioned factors, the environmental conditions, diet, and feeding behavior of corvids, combined with a high density of infected animal, human, and feline populations, could play a significant role in crow contamination. Numerous studies conducted in the last decade in Romania targeting humans, domestic definitive and intermediate hosts, as well as small wild mammals, most of which are food sources for opportunistic omnivorous corvids, support this hypothesis. Most national studies on toxoplasmosis were performed in the moist, warm, and low-altitude continental biogeographical region [65], including Central and North-Western Romania, where high prevalence levels are revealed (Figure 2 and Figure 3).

It is found that the high seroprevalence recorded in cats, the definitive host (62.9%) [66], correlates with the values in humans (35.9%) [67] and other intermediate hosts [68,69,70], including corvids. If the increased seroprevalence in humans is explained by the harmful eating habits of consuming less heat-sterilized animal products, the leading cause in domestic intermediate hosts could be the increased level of contamination of various environments (pastures, food storage) with *T*. *gondii* oocysts. In corvids, however, their omnivorousness and access to numerous contamination sources are likely the causes of the infection’s increased prevalence.

The comparative analysis of the *T*. *gondii* infection prevalence in corvids, worldwide and nationally detected, demonstrates a more or less pronounced heterogeneity, influenced by the previously mentioned factors. The direct role of these birds as sources of human contamination is almost non-existent. However, it is necessary to fill in the information gaps regarding the prevalence of *T*. *gondii* in bird populations globally, as some species are directly involved in public health. In addition, it is necessary to implement specific measures to prevent environmental contamination. Related to corvids, it is crucial to raise public awareness to collect the corpses of these birds, which can be a source of contamination for cats or other intermediate hosts. This will minimize the impact of *T*. *gondii* on environmental pollution and animal welfare [6].

## 5. Conclusions

Our findings, a high seroprevalence along with the detection of *T*. *gondii* DNA in a (limited) number of crows, support the idea that corvids, through their feeding habits and ecological interactions, are indeed sentinels. This suggests they may potentially contribute to the environmental circulation of *T*. *gondii*. To our knowledge, this is the first study describing *T*. *gondii* infection in corvids in Romania.

Overall, this work highlights the importance of the continuous surveillance of wild intermediate hosts. Increased public awareness about environmental contamination risks and targeted outreach campaigns are essential to reduce the impact of *T*. *gondii* on humans, domestic animals, and wildlife.

Future studies should focus on clarifying the epidemiological chains in which *T*. *gondii* is involved by standardizing surveillance techniques and diagnostic methods; this could improve our understanding of the parasite’s distribution and transmission dynamics in bird species.

## Figures and Tables

**Table 1:** Distribution of analyzed samples among five different corvid species.

Species		Total Number of Birds	Examined Using	
			MAT	PCR
jackdaw	*Coleus monedula*	52	52	52
rook	*Corvus frugilegus*	195	195	106
hooded crow	*Corvus cornix*	39	39	39
magpie	*Pica pica*	40	40	40
jay	*Garrulus glandarius*	7	7	7
Total		333	333	244

**Table 2:** Prevalence of *T*. *gondii* infection in corvids with MAT (≥1:24) and PCR.

Species	MAT			PCR		
	Frequency (n/N)	Prevalence (95% CI)	*p*-Value	Frequency (n/N)	Prevalence (95% CI)	*p*-Value
*C. monedula*	5/52	9.6 (4.2–20.6)	**0.0001**	0/52	0 (0.0–6.9)	**0.75**
*C. frugilegus*	44/195	22.6 (17.3–28.9)		1/106	0.9 (0.2–5.2)	
*C. cornix*	11/39	28.2 (16.5–43.8)		1/39	2.6 (0.5–13.2)	
*P. pica*	5/40	12.5 (5.5–26.1)		1/40	2.5 (0.5–12.9)	
*G. glandarius*	0/7	0 (0.0–35.4)		0/7	0 (0.0–35.4)	
**Total**	**65/333**	**19.5 (15.6–24.1)**		**3/244**	**1.2 (0.4–3.6)**	

**Table 3:** Prevalence of *T*. *gondii* infection in corvids with MAT dilutions.

MAT	*C. monedula*	*C. frugilegus*	*C. cornix*	*P. pica*	*G. glandarius*	Total
1:24	5 (9.6)	44 (22.6)	11 (28.2)	5 (12.5)	0	65 (19.5)
1:48	7 (13.5)	26 (13.3)	11 (28.2)	14 (35.0)	1 (14.3)	59 (17.7)
1:96	1 (1.9)	4 (2.1)	1 (2.6)	1 (2.5)	0	7 (2.1)
1:192	0	3 (1.5)	0	0	0	3 (0.9)

**Table 4:** Populations of corvid species in this study.

Species		World Population * (2012–2018)	European Population **	National Population ***
Western jackdaw	*Coloeus monedula*	38–84 **−**	9–20	320–550
Rook	*Corvus frugilegus*	54–95 ↓	8–14	150–200
Hooded crow	*Corvus cornix*	54–91.7 **−**	8–17	150–320
Eurasian magpie	*Pica pica*	64–104 **−**	7.5–19	500–1200
Eurasian jay	*Garrulus glandarius*	30–65 **−**	7.5–14	250–500

* Number of mature individuals (millions of specimens), ** Millions of pairs, *** Thousands of pairs, − stable, ↓ decreasing.

## Data Availability

All data may be shared and should be requested from the corresponding author.
